# Effects of Dietary Supplementation of Conjugated Linoleic Acids and Their Inclusion in Semen Extenders on Bovine Sperm Quality

**DOI:** 10.3390/ani11020483

**Published:** 2021-02-12

**Authors:** Mohammed S. Liman, Vittoria Franco, Claudia L. Cardoso, Valentina Longobardi, Bianca Gasparrini, Matthew B. Wheeler, Marcello Rubessa, Giulia Esposito

**Affiliations:** 1 Department of Production Animal Studies, Faculty of Veterinary Sciences, University of Pretoria, 0110 Pretoria, South Africa; limanms69@gmail.com (M.S.L.); dracardoso@gmail.com (C.L.C.); 2 Niger State Livestock and Fisheries Institute, Ministry of Livestock and Fisheries Development, 920001 Niger State, Nigeria; 3 Dipartimento di Medicina Veterinaria, Università degli Studi di Napoli Federico II, 80137 Naples, Italy; vittoriafranco1@gmail.com (V.F.); longobardivalentina@gmail.com (V.L.); bgasparr@unina.it (B.G.); 4 Department of Animal Sciences, University of Illinois, Urbana, IL 61801, USA; mbwheele@illinois.edu (M.B.W.); rubessa@illinois.edu (M.R.); 5 Department of Animal Sciences, Faculty of AgriSciences, Stellenbosch University, 7600 Stellenbosch, South Africa; 6 RUM&N Sas, 42123 Reggio Emilia, Italy

**Keywords:** CLA, semen evaluation, IGF-I, flow cytometry

## Abstract

**Simple Summary:**

Suboptimal fertility in males accounts for about two-thirds of infertility cases, thus being of serious concern for the dairy industry, where optimal fertility is fundamental for farm profitability. Although genetic defects responsible for subfertility have been identified, the role of seminal compounds on fertility remain unclear. Feeding rumen-protected isomers of conjugated linoleic acid (CLA) to dairy cows reportedly enhances circulating insulin-like growth factor I (IGF-I) levels. In breeding bulls, the IGF-I concentration in seminal plasma has been positively correlated with fertility rates. Therefore, the objective of the study was to evaluate the effect of dietary CLA supplementation and of their inclusion to the semen extender on bovine semen quality and freezability.

**Abstract:**

Isomers of conjugated linoleic acid (CLA) enhances circulating insulin-like growth factor I (IGF-I) levels. Furthermore, fertility rate of breeding bulls is positively correlated to seminal plasma IGF-I concentration. Our objective was to evaluate the effect of dietary CLA supplementation and inclusion to the semen extender on bovine semen quality and freezability. Fourteen bulls, randomly assigned to control (CTL) and CLA (50 g/day) groups, were supplemented for 10 weeks. Samples were collected at Weeks −2 (before supplementation), 0, 4, 6 (during supplementation), 10, and 11 (after supplementation). Blood and seminal plasma were analyzed for IGF-I; the ejaculates were frozen in the following subgroups: CTL (no addition to semen extender), CLA *c*9, *t*11 (50 µM), CLA *c*9, *t*11 (100 µM), CLA *t*10, *c*12 (50 µM), CLA *t*10, *c*12 (100 µM), and CLA mix (50 µM each of CLA *c*9, *t*11 and CLA *t*10, *c*12). Sperm motility, morphology, viability, mitochondrial membrane potential, and reactive oxidative species were assessed. CLA supplementation decreased ejaculates’ total volume, increased sperm concentration, beat cross frequency, and decreased oxidative stress; it also increased plasma and seminal plasma IGF-I levels compared to the CTL. The inclusion of CLA *c*9, *t*11 100 µM and CLA mixture in the extender increased live spermatozoa percentage post-thawing compared to other groups. Our results show a beneficial effect of CLA supplementation on semen quality; however, further studies evaluating fertilization rates are necessary to corroborate the results.

## 1. Introduction

In the cattle industry, suboptimal fertility in males accounts for about two-thirds of infertility cases, thus being of serious concern for the livestock industry [1,2,3]. Although genetic defects responsible for subfertility have been identified [1], the role of seminal compounds on fertility remain unclear [4,5,6]. Recent research has proved that metabolic hormones may improve semen fertility [7,8]. In particular, insulin-like growth factor-I (IGF-I) seems to play a significant role in differentiation, proliferation and steroidogenesis, and its derangement may be involved in male infertility [7,9,10]. In fact, IGF-I and insulin-like growth factor II (IGF-II), which are produced in the liver, have been identified in the testicles [11]; they are secreted by Leydig and Sertoli cells [11,12], and receptors for IGF-I have been reported in the spermatogonia, spermatocytes, spermatids, and spermatozoa [13,14]. Furthermore, studies indicate the possible role of IGF-I on sperm viability and motility in mouse [15], cattle [14], and buffalo [16,17]. In particular, the IGF-I concentration in the seminal plasma of healthy breeding bulls and stallions was positively correlated with fertility rates [10]. Selvaraju et al. [17] suggested that IGF-I might maintain motility through its antioxidant effect. In vitro studies also proved that the addition of IGF-I stimulates spermatozoa motility in bovine samples [14]. This is achieved by the activity of tyrosine kinase of the insulin-like growth factor I receptor (IGF-IR) and its ligand IGF-I present in the seminal plasma. It was speculated that the IGFR-IR and its ligand system are suspected to be involved in the signal transudation leading to sperm capacitation and acrosome exocytosis [18]. Blood concentration of IGF-I has been related to the energy balance of the animals [19] and to their reproductive performance [20,21]. Selvaraju et al. [22] showed that, by manipulating the diet of Nellore rams, IGF-I levels in seminal plasma increased with a positive effect on sperm quality.

In vivo studies have indicated that feeding isomers of CLA supplemented diets increases plasma levels of insulin-like growth factor (IGF-I) and IGF binding protein 3 (IGFBP3) in dairy cows [23,24]. However, very few studies investigated the effects of conjugated linoleic acids (CLA), especially the isomers *cis*-9, *trans*-11 and *trans*-10, *cis*-12 on male reproductive performance. Mehrdad et al. [25] evaluated the effects of dietary supplementation of CLA on reproductive performance in male mice. The study showed that CLA had significant effect on secondary sexual activity; the authors also speculated that CLA may have influenced spermatogenesis.

To our knowledge, there is only one study on the effects of dietary supplementation of CLA on fresh and post-thaw sperm quality on Holstein bulls [26], reporting little positive effect on both fresh and post-thaw semen quality such as fresh progressive motility and sperm velocity. On the other hand, few studies, with controversial results, have been published on the effects of CLA as possible semen protectants during the freezing process. In a recent study [27], the *cis*-9, *trans*-11 and *trans*-10, *cis*-12 isomers of CLA were added at 50, 100, and 150 µM into the cryopreservation media, with no clear advantages observed on the post-thaw bovine sperm integrity and functionality. In another study, CLA of *trans*-10, *cis*-12 isomer was added at 50 µM into cryopreservation media causing no advantages on the post-thaw boar sperm viability and integrity [28].

Our hypothesis was that, by supplementing the isomers *cis*-9, *trans*-11 and *trans*-10, *cis*-12 of the conjugated linoleic acid to the bulls, plasma and seminal plasma IGF-I levels might increase, thus improving sperm quality. Furthermore, by adding the CLA isomers to the semen extender, their antioxidant action would have minimized cryodamage therefore providing better quality sperm post-thawing. Therefore, the objectives of our study were: (1) to evaluate the effect of dietary CLA supplementation in bulls, on blood plasma and seminal plasma IGF-I levels and on post-thaw sperm quality; (2) to evaluate the effect of the inclusion of the isomers of the CLA at different concentration on post-thaw sperm quality; and (3) to evaluate a possible interaction between treatments and which approach is the more performing.

## 2. Materials and Methods

### 2.1. Experimental Location and Ethical Clearance

Experimental procedures were approved by the University of Pretoria Research Animal Committee (Project # V087-15). Samples collection was conducted during summer (November 2015 to March 2016) at an authorized commercial semen station located in the Free State, South Africa (29°05.70′ S, 026°20.42′ E). The semen station regularly hosts 20 dairy bulls and 4 beef bulls. All the animals were tested disease-free prior to be introduced.

### 2.2. Animals and Treatments

Fourteen adult dairy *Bos taurus* bulls (Jersey), complying with the minimum standards of frozen semen production of the enterprise (minimum semen production of 20 × 10^6^ sperm mL^−1^, >60% motility fresh semen, >50% live/motile sperm post-thawing, <30% morphological abnormalities, and >20% live sperm after 2 h stress test at 34 °C) were blocked by age (72 ± 18 months), BW (600 ± 200 kg), and BCS (3–3.5 in a 5-point scale), and randomly allocated to the two dietary treatments (refer to Supplementary Materials for more details about sample size): control (*n* = 7; CTL) and CLA (*n* = 7).

The animals were kept in individual sawdust bedded pens (3 × 3 m) with *ad libitum* water and fed twice daily at 900 and 1600 with a semi-complete pellet (Reprotech Mature *bull,* AFGRI-Animal feeds^®^, Centurion, South Africa) at 5% of body weight, and with alfalfa hay at 2% BW.

To maintain isolipidic and isoenergetic diets in both groups, during the morning feeding, each bull, according to the group allocation, received either a top-dressed supplementation of 50 g of rumen-protected conjugated linoleic acids (CLA group; Lutrell pure^®^ BASF, Ludwigshafen, Germany, providing 5 g of *trans*-10, *cis*-12 and *cis*-9, *trans*-11 CLA isomers) or a placebo composed by an equivalent amount of rumen inert fatty acids as control (CTL group; 40 g/day of Energy Booster 100; Milk Specialties Global, Eden Prairie, MN; containing 98.7% Free Fatty acid; 2.5 % C12:00; 28% C16:0; 45% C18:0; 8.3% C18:1; 1.5% C18:2 and 0.1% C18:3). Both supplements were mixed with 50 g molasses meal (Molatek, Ltd., Malelane, South Africa) to increase their palatability. Chemical composition of feeds and molasses meal are shown in Table 1.

The study lasted fourteen weeks: 2 weeks pre-supplementation (Weeks −2 and −1); a supplementation period of 10 weeks (Weeks 0–9) to cover a complete spermatogenic cycle [29]; and two weeks post-supplementation (Weeks 10 and 11).

### 2.3. Sample Collection

From each bull, blood and semen samples were collected twice a week during the following periods: Week −2 (pre-supplementation); Weeks 0, 4, and 6 (supplementation); and Weeks 10 and 11 (post-supplementation). The samples collected during the pre-supplementation period were analyzed to set the baseline levels for all the parameters investigated; during the supplementation period (Week 0–9), samples were collected at the beginning, during, and towards the end of the supplementation in order to cover different phases of the spermatogenic cycle; samples collected at Weeks 10 and 11 were analyzed to evaluate any possible carryover effect of the dietary treatment. Ejaculates were collected via artificial vagina as described by Chenoweth [30]. Soon after, blood samples were collected via coccygeal venipuncture into a 10 mL Vacutainer tube (BD Vacutainer^®^, Franklin Lakes, NJ, USA) containing EDTA anticoagulant.

### 2.4. Sample Processing

Immediately after collection, blood was centrifuged (Avanti j-e Beckman coulter) at 3000 rpm for 15 min and the resulting plasma collected and stored into 1.5 mL microcentrifuge tubes (Eppendorf^®^, Hamburg, Germany, Safe-Lock microcentrifuge tubes) at −20 °C for analyses. One milliliter of freshly collected semen was taken from each sample and kept on ice for a minimum of 15 min for the extraction of seminal plasma, as described by Macpherson et al. [10]. Briefly, the semen was centrifuged at 2000 rpm for 10 min at 20 °C; supernatant was collected and placed in a new tube and maintained on ice for 5 min, whereas the pellet was discarded. Chilled supernatant samples were centrifuged again at 2000 rpm for 30 min, and the new undiluted supernatant was filtered using 0.22-μM nylon syringe filters (MillexGV, Durapore^®^, Merk Pty, Ltd., Modderfontein, South Africa) to remove any remaining sperm cells. Filtered supernatant (the seminal plasma) was stored into 1.5 mL microcentrifuge tubes at −80°C. The remaining ejaculate was kept in water bath at 35 °C for about 3–5 min until determination of volume (measured using graduated glass conical tubes) semen concentration, pH, and volume of extender to be used (determined by SDM 6, Minitube^©^, Verona, WI, USA). Sperm motility (mass and individual) was estimated using a phase-contrast microscope (Nikon^©^, Centurion, South Africa).

Immediately after analyses, the semen was extended to a final concentration of 20 million/mL. Triladyl^®^ (Minitube^©^, Verona, WI, USA) was used as the base for the semen extender as normal routine of the semen station. It was formulated the day before sampling to have a pH of 6.8 and stored at 5 °C. The extender was composed of 250 mL Triladyl^®^, 250 mL pure egg yolk, 750 mL of distilled water, and supplemented with different CLA isomers and concentrations as follow:

1. Control: No supplementation into the Triladyl^®^ base extender
2. CLA *c*-9, *t*-11-50: Triladyl^®^ base extender supplemented with 50 µM of CLA *c*-9, *t*-11
3. CLA *c*-9, *t*-11-100: Triladyl^®^ base extender supplemented with 100 µM of CLA *c*-9, *t*-11
4. CLA *t*-10, *c*-12-50: Triladyl^®^ base extender supplemented with 50 µM of CLA *t*-10, *c*-12
5. CLA *t*-10, *c*-12-100: Triladyl^®^ base extender supplemented with 100 µM of CLA *t*-10, *c*-12
6. CLA mix: Triladyl^®^ base extender supplemented with a mixture of the two isomers at 50 µM each

Thus, for each dietary treatment (CTL and CLA), the semen was frozen into 6 freezing treatments according to the CLA isomer and its concentration added to the semen extender, thus obtaining 12 “dietary × treatment” groups.

The CLA isomers *cis*-9, *trans*-11 and *trans*-10, *cis*-12 (>90% pure; Nu-Chek Prep Inc., Elysian, MN, USA) were conjugated to FA-free BSA (Sigma-Aldrich Co.; St. Louis, MO, USA) in a 4:1 ratio to prepare 4.5 mM BSA-CLA stocks using a method adapted from Keating and collaborators [31]; further dilutions were made in the extender. To avoid any confounding effect due to the addition of the FA-free BSA to the treatment groups, FA-free BSA was added also to the control group. The concentrations of CLA used in the extender were based on previous studies on addition of fatty acids in semen cryopreservation media [27,32].

The amount of ejaculate needed to obtain a final concentration of 20 × 10^6^ sperm mL^−1^ previously calculated was added to each group. Thus, for each dietary treatment (CTL and CLA), each semen sample collected from every bull was frozen into 6 subgroups (with different treatment). The extended semen was then gradually cooled to 4 °C for 3 h in a cold room. Subsequently, the semen was loaded in previously labeled (according to bull number, dietary treatment, and treatment in the extender) 0.25 mL Cassou straws^®^ using of a straw filling and sealing machine (IMV, MPP Uno, 13017/0000, Minitube^©^). Sealed straws were kept horizontally on trays and transferred into an automatic programmable biological cell freezer (ice cube 14 M, Minitube^©^) and frozen following the standard protocol. Briefly, the temperature decreased at a programmed rate of −3 °C/min from +4 to −10 °C; −40 °C/min from −10 to −100 °C; and −20 °C/min from −100 to −140 °C. Then, the straws were plunged into liquid nitrogen (−196 °C) and stored for later analysis.

### 2.5. Analyses

For each bull, after freezing, the samples collected twice weekly were pooled, to eliminate the effect of daily variability, thus obtaining one pooled sample/bull/week for each sample type (e.g., blood plasma and seminal plasma). Furthermore, to eliminate the straw effect, two straws for each collection were used. All analyses were conducted by a technician unaware of the treatment groups.

### 2.6. IGF-I Levels in Blood and Seminal Plasma

Blood and seminal plasma were analyzed for insulin-like growth factor-I (IGF-I) levels using a Bovine IGF-I ELISA Kits (CUSABIO^®^, Ltd., Houston, TX, USA) following the manufacturer guidelines.

### 2.7. Sperm Evaluation

Semen straws were thawed in water bath at 37 °C for 30 s; the straws were then dried with a paper towel and the semen was subsequently transferred to a microcentrifuge tube, previously heated, and kept incubated at 37 °C.

### 2.8. Sperm Motility

For the assessment of sperm motility, 3 replicas of 10 μL of thawed semen was loaded into a pre-warmed (38 °C) chamber slide (20 mm; Leja^®^4D Products B.V, Nieuw-Vennep, The Netherlands), with cover slip applied; sperm motility was assessed using a Computer-Assisted Sperm motility analyzer (CASA; IVOS Version 12.3: Hamilton-Thorne Biosciences, Beverly, MA, USA) set on the animal motility program preadjusted for the analysis of bovine sperm, as reported by Peres Companholi et al. [33]. These settings allowed capturing 60 frames per second and storing 30 frames to obtain values, compiled across all fields examined, for >500 sperm.

The parameters evaluated were total motility (MT; %), progressive motility (PROG; %), average path velocity (VAP; μm/s), straight line velocity (VSL; μm/s), curvilinear velocity (VCL; μm/s), lateral displacement amplitude (ALH; μm/s), beat cross frequency (BCF; Hz), straightness (STR; %), and linearity (LIN; %).

### 2.9. Sperm Morphology

For each bull, dietary treatment, and addition of CLA into the extender, post-thaw semen underwent sperm morphology evaluation. Hence, 10 µL of thawed semen were placed on a pre-warmed slide (38 °C) and mixed with the same amount of eosin/nigrosine stain as described by Chenoweth [30]. For each sample, 3 replicas were analyzed. The percentage of total sperm with morphological defects was determined by counting a minimum of 100 sperm per slide using a phase-contrast microscope (AX10 Zeiss©, Johannesburg, South Africa) at a magnification of 100× (oil immersion).

The identified defects of each sperm were recorded, including those with double defects. According to Barth and Oko [34], these defects were divided into those which affect the head (teratoids, double, macrocephalic, microcephalic, rooled/crested head, pyriform, tapered/narrow head, diadem/nuclear crater, narrow base, abnormal base, other abnormal head shapes, and abnormal loose head) and those which affect acrosome or tail (knobbed acrosome, stump tail, pseudo-droplets, mal-positioned mitochondria, cork screw, dag, other mid-piece defects, coiled principle piece, proximal droplets, mid-piece reflex, normally shaped loose heads, fractured flagellum, distal droplets, damaged acrosome, bent mid-piece, and bent principle piece). The counts were calculated for the varied defects and recorded on a data capture sheet for bull sperm morphology adopted from Nöthling and Irons [35].

Percentage of morphologically normal spermatozoa were calculated following Nöthling and Irons [35] as: 100 (number of sperm cells counted) − *n* (number of sperm cells with one or more nuclear (head) defects + number of spermatozoa with one or more acrosome or tail defects − spermatozoa with nuclear defects as well as acrosomal or tail defects).

### 2.10. Sperm Viability, Mitochondrial Membrane Potential and Oxidative Stress

Flow cytometric analyses were conducted on the post-thawed semen using flow cytometry FC 500© Beckham Coulter (Brea, CA, USA) equipped with a 488 nm argon-ion laser. Red fluorescence was detected via a FL3 band-pass filter (610 nm) and green fluorescence was evaluated via fluorescence channel (FL1) band-pass filter (525 nm). In total, 50,000 sperm cells were analyzed. The flow machine was gated, and a test run was conducted to optimize the technique, minimize the background noise, and ensure repeatability [36,37,38]. Thawed semen was prepared and added to Phosphate-buffered saline (PBS) solution according to staining method suggested by the manufacturer of each kit. The analyses were carried out in duplicate to enable repeatability, and the followings were evaluated: sperm viability, mitochondrial membrane potential, and oxidative stress.

For the viability test, a mixture of propidium Iodide (PI) and SYBR-14 dyes (LIVE/DEAD sperm viability kit L-7011, Molecular probes-Invitrogen) was used. The samples were prepared following the manufacturer instructions. Briefly, the master mix (480 μL of hepes/Bovine Serum Albumin buffer, 2.5 μL of SYBR-14 (1:50 dilution), and 2.5 μL of Propidium Iodide) was made. Fifteen microliters of pooled thawed semen maintained at 34 °C was added to 485 μL of master mix, which was constantly kept away from sunlight. The mixture was then vortexed and incubated at 37 °C for 15 min. After the incubation time, 500 μL of prepared semen were loaded into tubes (12 × 75 mM, Blue Test Tube, FC 500© Beckham coulter Life Science, Johannesburg, South Africa) and run into the flow cytometer to assess the number of live and dead sperm cells. Membrane-permeant SYBR^®^-14 nucleic acid stain labels live sperm with green fluorescence, and membrane-impermeant propidium iodide labels the nucleic acids of membrane-compromised sperm with red fluorescence. Mitochondrial membrane potential was measured using the JC-1 mito-tracker (Molecular probes-Invitrogen, Thermo Fisher Scientific, Waltham, MA, SA) and 10% Dimethyl sulfoxide (DMSO; Sigma Aldrich, Merk Pty, Ltd., Modderfontein, South Africa). The dyes were brought to room temperature and 490 μL of master mix (prepared with 9 mL of Dulbecco’s Phosphate-buffered saline solution (DPBS) and 82 μL of dye to have a concentration of 3 mM JC-1 in DMSO) were mixed with 10 μL of pooled thawed-semen maintained at 34 °C and kept away from light. Thereafter, the mixture was vortexed and incubates for 15 min at 37 °C. It was then washed once with 500 μL of PBS, centrifuged at 2000 rpm for 5 min (Thermo Scientific-Heraeus Freso 21 microcentrifuge), and the supernatant was removed. Furthermore, 500 μL of PBS were added and the samples were run on flow cytometer to assess sperm membrane mitochondrial potential. The JC-1 dye exhibits potential-dependent accumulation in mitochondria, indicated by a fluorescence emission shift from green (~529 nm) to red (~590 nm). Consequently, mitochondrial depolarization is indicated by a decrease in the red/green fluorescence intensity ratio. The potential-sensitive color shift is due to concentration-dependent formation of red fluorescent J-aggregates. Alteration of membrane potential indicates intact or disrupted mitochondria.

Oxidative stress was measured by means of measuring reactive oxygen species (ROS) using the 2,7-diclorodihydrofluorescein diacetate (H2DCFDA) dye (Thermo Fisher Scientific, Waltham, MA, USA) following the manufacturer instructions. The (H2DCFDA) is a cell-permeable nonfluorescent probe which is de-esterified intracellularly and turns to highly fluorescent 2′,7′-dichlorofluorescein upon oxidation. The dye was diluted and mixed with DPBS to make a master mix (9 mL of DPBS and 170 μL of dye); 490 μL of dye was added to 10 μL of pooled thawed-semen maintained at 34 °C and kept away from sunlight. The mixture was vortexed and incubated for 30 min at 37 °C. Thereafter, it was washed once with 500 μL of PBS and centrifuge at 2000 rpm for 5 min (Thermo Scientific-Heraeus Freso 21 microcentrifuge). Supernatant was removed and 500 μL of PBS were added, vortexed, and the samples run in flow cytometer to assess the level of ROS. To simultaneously differentiate living from dead cells propidium iodide (PI, Molecular Probes Inc., Thermo Fisher Scientific, Waltham, MA, USA; final concentration, 9.6 mM) was added to H2 DCFDA-treated sperm.

### 2.11. Experimental Design and Statistical Analyses

The study lasted fourteen weeks with two weeks pre-supplementation (Weeks −2 and −1), 10 weeks of supplementation (Weeks 0–9), and 2 weeks post-supplementation (Weeks 10 and 11). More details about the samples size are provided in the Appendix A.

Data were analyzed with SAS 9.3 software (SAS Institute Inc Cary, NC, USA, 2012). Weekly mean of IGF-I in blood and seminal plasma, and the morphology results were analyzed by analyses of variance (ANOVA) for repeated measures using a mixed model (PROC MIX, SAS 9.3; SAS Institute, Cary, NC, USA) with a mathematical model where treatment (trt: CTL and CLA), time (week), and the interactions between treatment and time were fixed effects, whereas the bulls were the random effect.

CASA and flow cytometric results were analyzed by analyses of variance (ANOVA) for repeated measures using a mixed model (PROC MIX, SAS 9.3, SAS Institute, Cary, NC, USA) with nested variable effects in a mathematical model where treatment (dietary treatments CTL and CLA), time (week), subgroup (CTL and different CLA isomers at different concentration into Triladyl^®^ extender), and their interactions were fixed effects, while the bulls were the random effect. Data obtained from Week -2 were used as covariate; significance was declared with *p* ≤ 0.05 and trend with 0.05 > *p* ≤ 0.10.

## 3. Results and Discussion

### 3.1. Effects of Dietary Treatments on Plasma and Seminal Plasma IGF-I Levels

**Figure 1:**
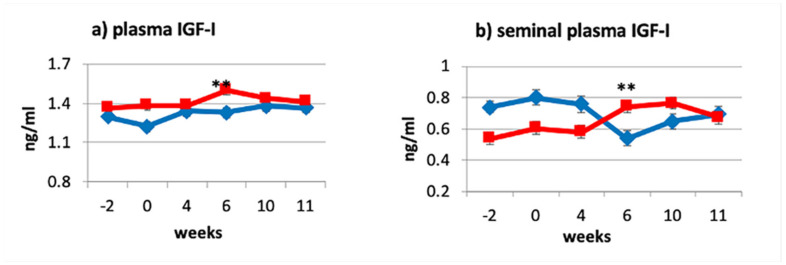
Effect of CLA dietary supplementation on bovine IGF-I levels in plasma (**a**) and seminal plasma (**b**) for the control (CTL♦) and CLA (■) group. Data are expressed as least square means (LSM) ± standard error (SE); ** significant levels trt × week: *p* ≤ 0.05.

A significant interaction trt × week was observed for the blood IGF-I concentration in the CLA supplemented bulls (*p* ≤ 0.01; Figure 1a) with significant differences observed by Week 6 (CLA 1.50 ± 0.04 ng/mL, CTL 1.33 ± 0.04 ng/mL) and disappearing after treatment (Weeks 10 and 11). Although, to the best of our knowledge, no other studies evaluated the effect of dietary CLA supplementation on circulating IGF-I levels in bulls, our results are in line with what has been observed in dairy cows [23,24].

As expected, the concentration of IGF-I in seminal plasma followed the same pattern as for the blood plasma (trt × week: *p* ≤ 0.01; Figure 1b).

The observed results support our hypothesis by which dietary supplementation of CLA would have increased seminal plasma IGF-I by increasing its blood levels. Although the mechanism of action of the CLA remains unclear, these results support the theory of Csillik et al. [39] which claim that the effect of CLA supplementation is independent of animal’s energy balance status. Furthermore, studies conducted on male growth hormone (GH) deficient dwarf rats proved that treating the rats with exogenous GH resulted in higher concentrations of IGF-I in blood plasma and seminal vesicle fluid compared with non-dwarf control rats [40]. Thus, we could speculate that CLA may play a role in the GH–GH receptor–IGF-I axis, increasing circulating levels of IGF-I. As speculated by Castañeda-Gutiérrez et al. [23], the effects of CLA for increasing plasma IGF-I may be mediated by subtle changes on hepatic sensitivity to insulin action that are specific to the trans-10, cis-12 CLA isomer. However, contributing to the statistical difference observed between groups is also the drop in IGF-I observed in the control group. Due to the study being conducted during summer, the drop, by Week 6, in IGF-I levels in the seminal plasma of the control animals could be attributed to the negative effect which, according to some researchers, heat stress has on circulating IGF-I levels [41,42] In fact, the average daily temperature for December, January and February was, respectively, 39, 41, and 37 °C, with Week 6, during which a drop in IGF-I levels has been recorded, falling in the hottest month. The drop in temperature following Week 6 may explain the increased level of IGF-I in Weeks 10 and 11. However, IGF-I concentration decreased only in seminal plasma levels but not in blood, supporting what has been affirmed by other researchers [41,43,44], based on which circulating IGF-I levels do not change during heat stress. Thus, it could be speculated that, although heat stress did not alter blood levels of IGF-I, it might have reduced the response of IGF-I receptors present in the male reproductive tract [14,18] and that the CLA diet mitigated this effect.

### 3.2. Effects of Dietary Treatments on Fresh Semen Characteristics

**Figure 2:**
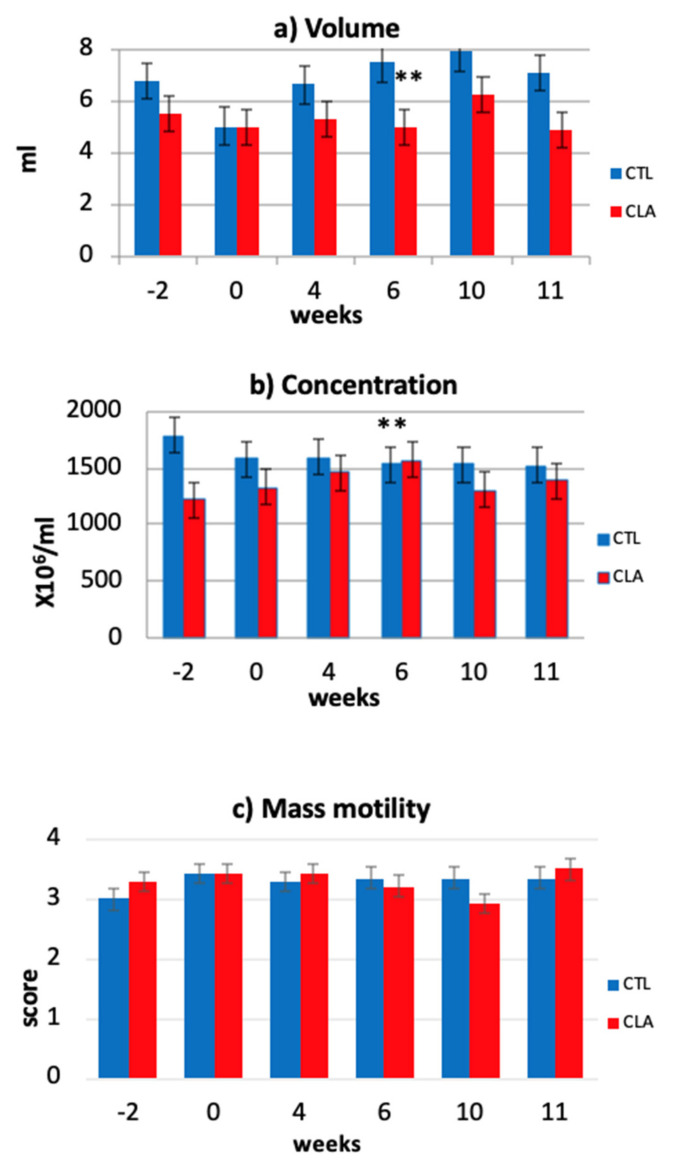

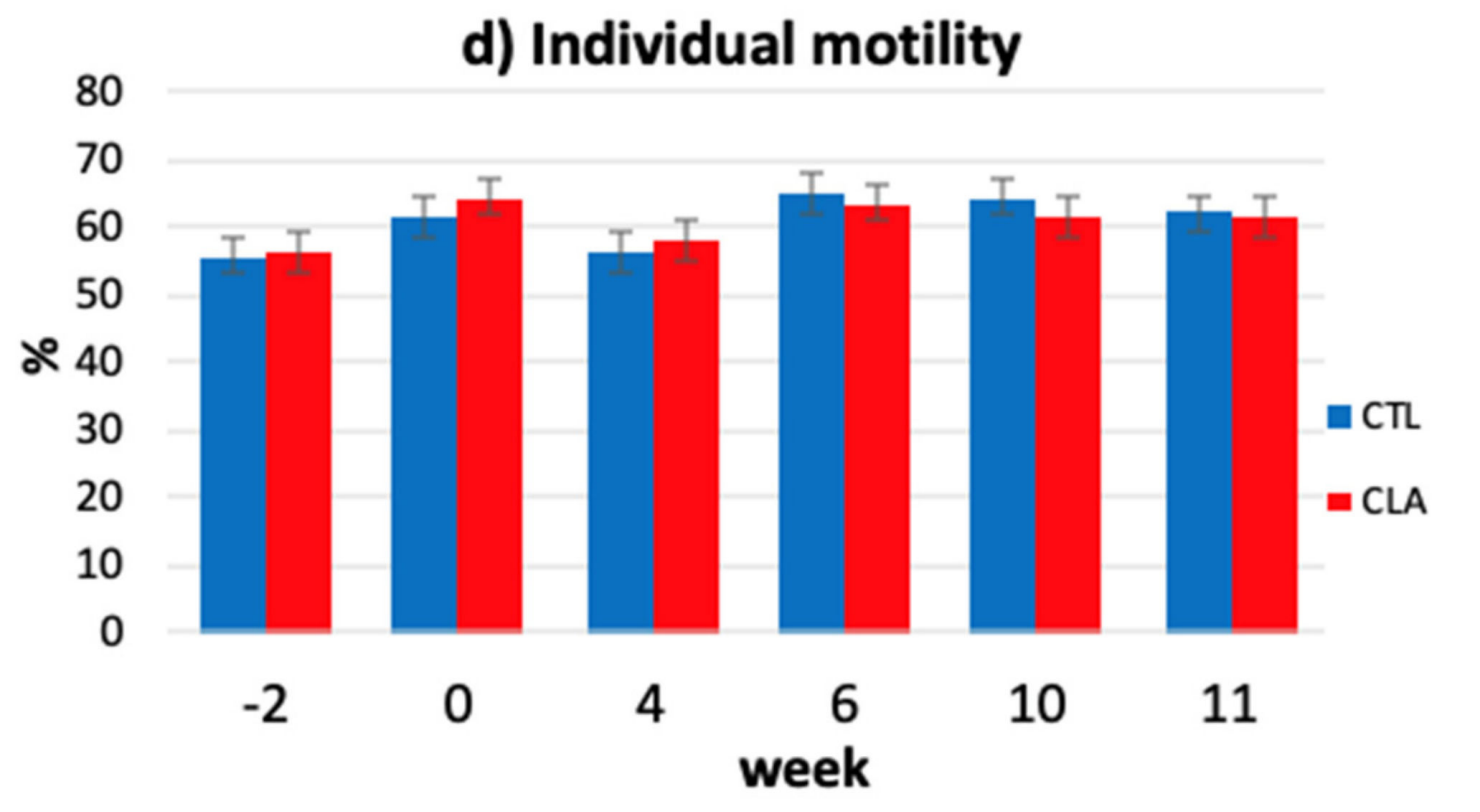
Effect of CLA dietary supplementation on fresh ejaculated bovine semen volume measured using graduated glass conical tubes (**a**); concentration determined by SDM 6, Minitube^©^ (**b**); and mass motility (**c**) and individual motility (**d**) estimated using a phase-contrast microscope (Nikon^©^) (LSM ± SE) for the control (CTL) and CLA group. ** significant levels trt × week: *p* ≤ 0.05.

Volume of the ejaculate significantly decreased during the supplementation period for the CLA group compared to the control (LSM ± SE: 6.83 ± 0.394 and 5.33 ± 0.394; trt × week *p* ≤ 0.02; Figure 2a) with volumes for the CLA bulls still within the normal range (0.5–12 mL) [45]. Conversely, semen concentration increased over time with a higher semen concentration in the CLA group compared to control group at Week 6 (1569.21 ± 156.28 × 10^6^; CTL: 1532.57 ± 156.28 × 10^6^; trt × week *p* ≤ 0.05; Figure 2b). For both parameters, there was no carryover effect of the treatment after supplementation with no differences observed between groups at Weeks 10 and 11 (post-supplementation). These results are in line with the results of Karimi et al. [26], who also observed a significant reduction in volume of the ejaculate in the CLA supplemented bulls compared to the control group (9.6 ± 0.9 and 12.4 ± 0.9 mL, respectively) and an inverse trend for sperm concentration. A possible explanation for the increase in sperm concentration could be given by the increased blood and seminal plasma levels of IGF-I. It is known, in fact, that this hormone, together with IGF-II and insulin has several effects on Sertoli cells, increasing proliferation, affecting steroidogenesis, and promoting spermatogonia differentiation into primary spermatocytes [11,46]. However, the effect of dietary CLA supplementation on the volume of the ejaculates is still unclear and needs further investigation. In contrast with what was observed by Tran et al. [47], but in line with what was observed by Karimi et al. [26], no significant differences were observed between groups for mass motility and individual motility (Figure 2c,d).

### 3.3. Sperm Morphological Defects

Dietary CLA supplementation did not affect the percentage of normal spermatozoa (CTL 82.75 ± 1.717; CLA 83.76 ± 1.717, *p*: 0.68) however, it recorded significant effects in different morphological defects, classified for simplicity, according to the stages of development in which they may occur (Tables S1–S5).

At Week 4, we recorded lower percentage for teratoid defect (CLA 0.23% ± 0.10, CTL 0.64% ± 0.10; trt × week *p*: 0.01) and crested head (CLA 0.11% ± 0.15, CTL 0.63% ± 0.15 trt × week *p*: 0.002) and a higher percentage of macrocephalic head defect (CLA 0.69% ± 0.12 vs. CTL 0.28% ± 0.12; trt *p* ≤ 0.01) for the CLA group compared to the control. The effects worn off by Week 10 (Table S1). During the early phase of meiosis, teratoid defects are characterized by severely underdeveloped spermatozoa, whereas microcephalic heads (abnormally small heads) originate from incomplete meiosis [34]. These defects (teratoid defect and microcephalic heads) may sometimes be the result of frequent semen collection in semen centers. Therefore, we hypothesized that CLA supplementation somehow limited the negative effects of frequent semen collection. Although pseudo-droplet and crested head defects are likely genetically inherited defects in Holstein breeds [34], it would appear that CLA dietary supplementation may have contributed to a reduction of the defects during supplementation. Narrow head defect was significantly higher in the CLA animals compared to control at Week 4 (CLA 0.58% ± 0.146, CTL 0.20% ± 0.146; trt × week *p*: 0.072) and become significantly lower by Week 11(CLA 0.25% ± 0.15, CTL 0.78% ± 0.15; *p* ≤ 0.01; Table S2).

A significant interaction trt × week was observed for bent midpiece and bent principle piece defects, with the CLA bulls presenting spermatozoa with a higher percentage of bent midpiece and bent principle piece defects at Week 4 (CLA 2.70% ± 0.34, CTL 1.56% ± 0.34; *p* ≤ 0.02; and CLA 0.90% ± 0.14, CTL 0.22% ± 0.14; *p* ≤ 0.05, respectively; Table S4). Although CLA dietary supplementation increased blood and seminal plasma levels of IGF-I, our results are in contrast with those of Garner and Johnson [48], who reported that dwarf rats treated with IGF-I presented spermatozoa with no mid-piece abnormalities (0%), compared to their control (0.04% ± 0.05).

An interaction trt × week was observed also for the loose head defects. In fact, although loose head defect was higher in CLA bulls compared to the control from the beginning of the study (CLA 1.29% ± 0.20, CTL 0.71% ± 0.20; *p* ≤ 0.04; two weeks prior to the supplementation), by the end of the supplementation period (Week 11), it was reduced by half (CLA 0.43% ± 0.10, CTL 0.14% ± 0.10; *p* ≤ 0.05). The CLA supplementation might have affected this parameter through increasing seminal plasma IGF-I levels. In fact, dwarf rats treated with IGF-I recorded lower percentage of loose head defects compared to their control [49]. Furthermore, the distal droplet defect recorded low percentage for CLA bulls (CLA 0.11% ± 0.15, CTL 0.79% ± 0.15; trt × week *p* ≤ 0.05) at Week 4. This could have resulted from the CLA increasing the levels of hemolytic factor produced by the vesicular glands [34].

For two or more morphological defects on a single spermatozoon (Table S5), double nuclear defect recorded significant low percentage at Week 4 in CLA bulls compared to the control (CLA 0.00% ± 0.03, CTL 0.12% ± 0.03; trt × week *p* ≤ 0.01).

None of the morphological parameters was affected by either the addition of CLA into the extender or their interaction with the dietary treatments.

Although CLA dietary supplementation did not increase the overall percentage of normal spermatozoa after freezing–thawing, as also observed by Karimi er al. [26], it did reduce the incidence of some defects, possibly through the effect that IGF-I has on cell morphology [49]. In fact, research has demonstrated that IGF-I treatment improves sperm cell morphology. Although Karimi et al. [26] did not report a detailed description of the sperm defects observed in their study, they did report that higher sperm abnormalities in the CLA group compared to the control one was recorded when analyzing fresh semen. The authors attributed these differences to possible increase of CLA in plasma membrane of spermatozoa that may have led to unfavorable changes in spermatozoa; however, they did not explain how these differences disappear after freezing–thawing [26]. Unfortunately, due to logistic impediments (with the semen station being 5 h drive from the laboratories), we were unable to perform morphological evaluation on the fresh samples to corroborate the observations made by Karimi et al.

Macpherson et al. [10] observed that stallions with higher concentration of plasma IGF-I levels recorded higher quantity of total IGFBP-2 in seminal plasma and higher percentage of morphologically normal sperm compared with those with lower plasma IGF-I levels (*p* = 0.05). Vickers and collaborators recorded a significant increase in both the morphologically normal spermatozoa and the number of motile spermatozoa in rats treated with IGF-I, with an improvement particularly for the head defects [49]. The authors speculated that these observations might have been a result of a direct effect of IGF-I on testicular function to improve the quality of spermatozoa. Although in our study we observed a reduction in percentage of some head defects, we did not observe any significant difference between the two groups in the total morphologically normal spermatozoa. Therefore, even though we observed an increase in IGF-I levels in blood and seminal plasma for the CLA bulls compared to the control, the results regarding sperm cell morphology do not allow us to draw conclusions on the possible effect of CLA dietary supplementation on sperm morphology or on its possible action via IGF-I.

### 3.4. Kinematic Parameters

**Figure 3:**
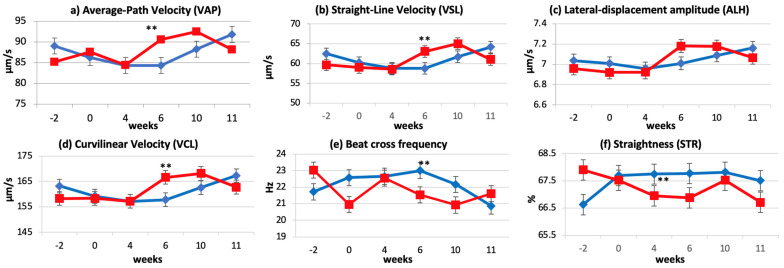
Effect of CLA dietary supplementation on post-thaw bovine semen CASA-derived kinematic parameters of: VAP (**a**); VSL (**b**); ALH (**c**); VCL (**d**); BCF (**e**); and STR (**f**). (LSM ± SE) for the control (CTL ♦), and CLA (■) group; ** significant levels trt × week as *p* ≤ 0.05; * trend trt × week at *p* ≤ 0.1.

Dietary supplementation of conjugated linoleic acid (CLA) had an effect over time on some kinematic parameters. In fact, by Week 6, the CLA group recorded a significant increase in VAP VSL, ALH, and VCL (trt × week, *p* ≤ 0.03; Figure 3a–d), a significant decrease in BCF (trt × week, *p* ≤ 0.05), and a trend for decreased STR (trt × week, *p* ≤ 0.09; Figure 3e,f) compared to the control group. No carryover effect of the supplementation was observed at Week 10 or 11. No differences between groups were observed for other CASA parameters (motile, rapid, total, and progressive motility). Even though CLA dietary supplementation significantly affected kinematic parameters, none of the parameters were affected by the addition of any of the CLA isomers to the Triladyl^®^ extender.

The kinematics parameters VAP, VCL, VSL, ALH, BCF, and STR are reported to be good fertility indicators and, therefore, they are commonly evaluated [50,51]. In bovines specifically, VCL, VSL, ALH, VAP, and progressive motility are reported to be highly correlated to fertility [52]. Our results are in contrast with those of Karimi and collaborators [26] on kinematics parameters of frozen–thawed semen. In fact, although they observed some effects on fresh semen, they could not detect significant differences between control and CLA groups in frozen–thawed semen, except for the lower total motility and BCF values at Week 8 in the control group and the decreased total motility, VCL, and ALH at Week 6 in the CLA group [26].

However, findings similar to what was observed in this study were reported in goats [53], rams [54], and boars [55] when polyunsaturated fatty acids (PUFA) were used as supplement; however, contrasting results were reported from studies conducted in Nalli-Ravi buffalo bulls [56] and Holstein bulls [57], where no correlation between dietary PUFA supplementation and sperm quality were observed. According to the studies of Breier and collaborators and Vickers et al. [40,49], the effects of seminal plasma IGF-I on sperm motility were mediated via secretion of IGF-I in the seminal vesicles and binding to the IGF-I receptor on developing sperm cells as the cells were mixed with vesicular fluid. Therefore, we could speculate that the CLA affects sperm motility via IGF-I receptors

### 3.5. Flow Cytometry Analyses

**Figure 4:**
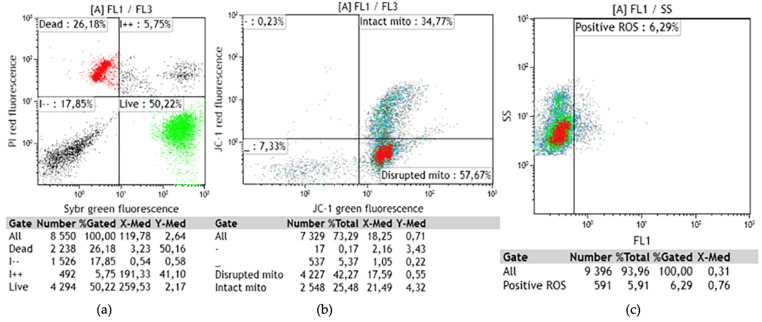
Flow cytometric forward scatter gated analysis on one sample of post-thaw bovine sperm quality for viability (**a**); mitochondrial membrane potential (**b**); and ROS (**c**) (LSM ± SE) levels. (**a**) SYBR-14 and PI viability stain. Red dots represent dead sperm, green dots represent viable or live sperm, out-gated red/green dots represent recently moribund or membrane damaged sperm, and unstained dots represent debris with low fluorescence. (**b**) JC-1 mitochondrial membrane potential: Populations of sperm with high mitochondrial membrane potential (intact) with fluorescence shift from green to red, and low mitochondrial membrane potential (disrupted) with fluorescence intensity shift from red to green. (**c**) ROS levels using 2, 7-diclorodihydrofluorescein diacetate (H2DCFDA) dye, which de-esterified and fluoresced upon oxidation.

Figure 4 shows the flow cytometric forward scatter gated analysis for viability (Figure 4a), mitochondrial membrane potential (Figure 4b), and oxidative stress (Figure 4c).

**Figure 5:**
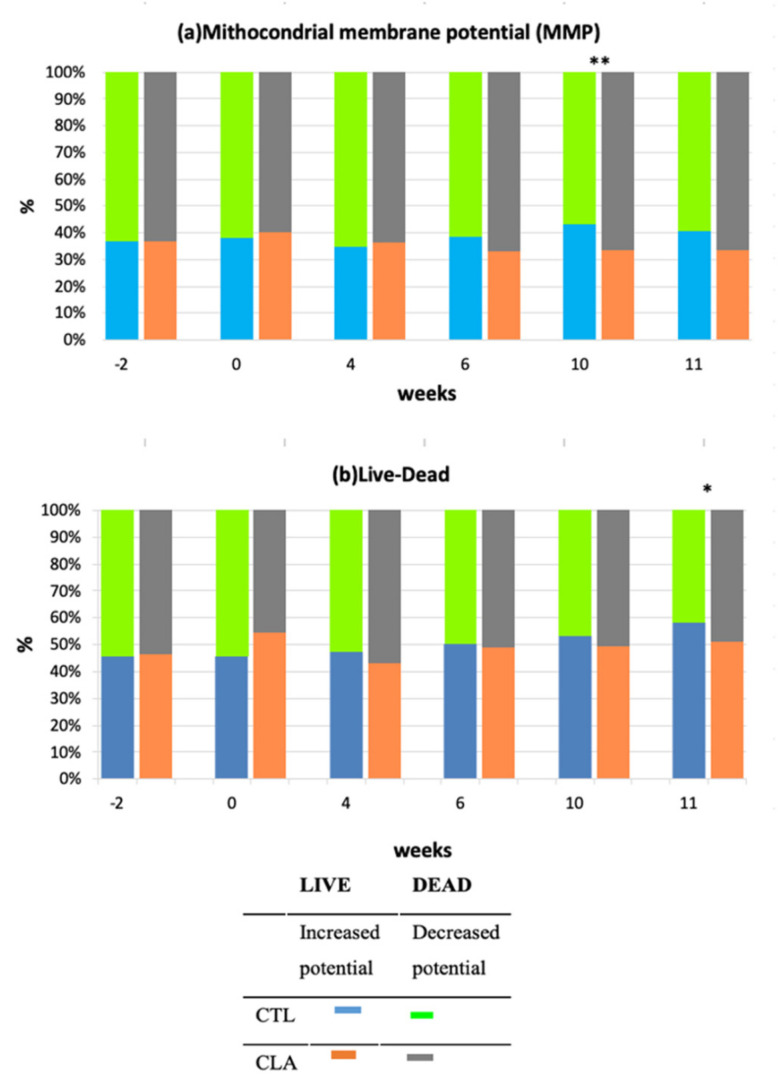
Effect of CLA supplementation on post-thawed bovine semen flow cytometer analysis of mitochondrial membrane potential (**a**) and live–dead sperm (**b**) for control (CTL) and CLA group (LSM ± SE). ** Significant levels *p* ≤ 0.05, and * a trend *p* ≤ 0.1.

Mitochondrial membrane potential significantly decreased in the CLA group compared to control (CLA: 64.44% ± 3.45; CTL: 54.60% ± 3.45; trt × week, *p* ≤ 0.05) at Week 10 (Figure 5a). A trend for increased percentage of dead spermatozoa (*p* ≤ 0.10) was also observed for CLA group compared to the control (CLA: 36.48% ± 2.86; CTL: 32.78% ± 2.86) at Week 11 (Figure 5b).

The decrease in mitochondrial membrane potential is an indication of initiation of cell apoptosis [57]. This could explain the trend for increased percentage of dead sperm at Week 11 for the CLA group. Although none of the other parameters affected by the treatment had any carryover effect after supplementation, we are unable to determine if the CLA supplementation had any effect on the mitochondrial membrane during the initial phase of spermatogenesis or if these findings are coincidental.

**Figure 6:**
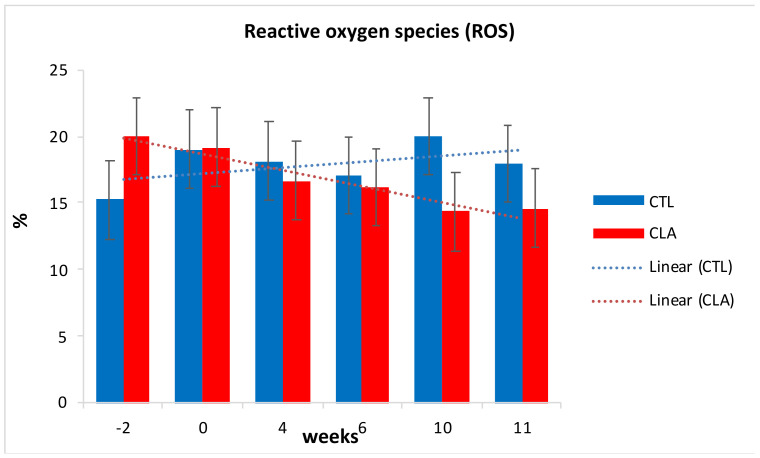
Effect of CLA dietary supplementation on post-thawed bovine semen flow cytometer analysis of percentage of reactive oxygen species ROS (LSM ± SE), for control (CTL) and CLA group.

A numerical decrease in percentage of ROS was observed for CLA group compared to control (*p* ≤ 0.1) from the beginning of supplementation until the end of supplementation (Figure 6). The lack of significance could possibly be explained by the high variability between samples justified by the high standard error. Although ROS are known for their damaging effects on spermatozoa, low levels and controlled concentration are essential for participation in signal transduction of sperm capacitation and acrosome reaction [11]. The decrease in ROS could be probably due to increase in protective action of GSH, as result of the CLA supplementation, as supported by other studies [58,59]. Neither an effect of CLA isomers addition to the Triladyl^®^ extender nor an interaction dietary treatment by semen extender treatments was observed for mitochondrial membrane potential and ROS.

**Figure 7:**
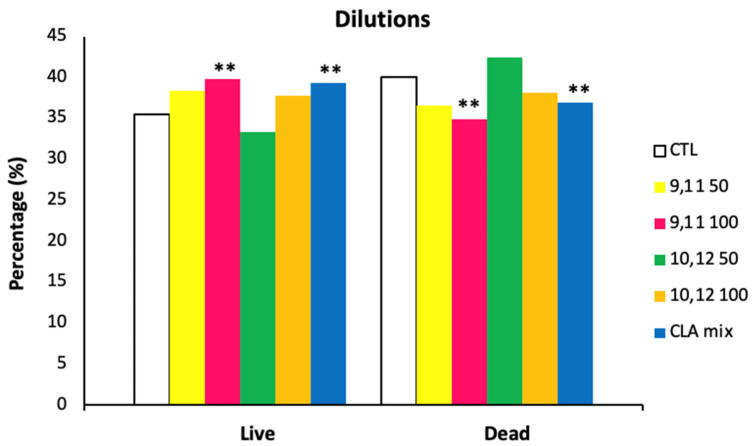
Influence of CLA supplementation and addition at different concentrations to Triladyl^®^ on post-thawed dairy bull semen for flow cytometer analysis of the percentage of live and dead sperm (LSM ± SE) for Control (CTL), CLA group. ** significant at 100 µM CLA *c*9, *t*11 and CLA Mix as *p* ≤ 0.05.

An effect of CLA isomers addition to the Triladyl^®^ extender was, however, noted on sperm viability with the addition of the *c*9, *t*11 isomer at 100 µM and of the CLA mixture resulting in a significantly higher percentage of live sperm compared to the control and the other treatments (LIVE: 39.10 ± 1.74% and 39.19 ± 1.74% for the *c*9, *t*11 at 100 μM and the CLA mix vs. LIVE: 36.20 ± 1.74%, 37.62 ± 1.74%, 34.45 ± 1.74%, and 37.40 ± 1.74% for the CTL, *c*9, *t*11 at 50 μM, *t*10, *c*12 at 50 μM, and *t*10 *c*12 at 100 μM; *p* = 0.006; Figure 7). However, no differences between the two groups (*c*9, *t*11 at 100 µM and the addition of the CLA mix) were observed. Furthermore, the interaction of dietary treatment by semen extender treatments had no significant effect.

This study, to the best of our knowledge, is the first demonstrating that 100 µM of the isomer *c*9, *t*11 and that the mixed isomers at 50 µM each improve the storage of spermatozoa irrespective of dietary supplementation with CLA. In fact, previous studies did not observe any difference between groups, on either post-thawed semen subjective analysis or the CASA indices [27,28]. However, none of the abovementioned studies investigated sperm viability to either corroborate or dismiss our findings.

Although our study showed a beneficial effect of CLA *c*9, *t*11 addition to the Triladyl^®^ extender at 100 µM and not at lower concentration (50 µM), we did not include CLA at a higher level than 100 µM to be able to determine the optimal level of inclusion.

## 4. Conclusions

Conjugated linoleic acids dietary supplementation could be an important strategy to enhance bovine reproductive performances as CLA positively affects sperm qualitative characteristics.

However, studies in males are very limited and most of them have been predominantly done with other PUFAs with only one study, to the best of our knowledge, on CLA supplementation on bovine semen.

The first part of the study proved the beneficial effects that CLA has on sperm motility and some morphological features, possibly by altering seminal plasma IGF-I levels and mitigating the negative effects that heat stress has on IGF-I. On the other end, the controversial results on flow cytometric analyses warrant further investigation. The study also highlighted that the effect of dietary supplementation was observed by Week 6 and did not have any carryover effect after supplementation. This suggests that CLA supplementation might play a role in the second half of the spermatogenic cycle.

The second part of this study aimed to reveal the effect of the addition of the CLA individual isomers, *c*9, *t*11 and *t*10, *c*12, into Triladyl^®^ extender on post-thaw bovine semen quality and their possible interaction with the CLA dietary supplementation. The addition of the isomer *c*9, *t*11 at 100 μM and the CLA mix isomers at 50 μM each recorded significant increased percentage of live sperms cells compared to the other groups. However, none of the other parameters observed showed any difference among groups and no interaction between the dietary treatment and the CLA addition into the semen extender was observed. Therefore, in accordance with previous studies, CLA addition to semen extender resulted in minor beneficial effects on semen quality; however, we were able to highlight which isomers, and at which concentration, are responsible for increase sperm viability.

In conclusion, the study highlighted the potential of CLA dietary supplementation in improving semen quality; however, to better understand the stage of the cycle in which CLA have effect, its mechanism of action, and the potential of CLA in mitigating the negative effects of heat stress, with the aim of identifying definite measures towards improving the semen quality, further studies conducted in hot and cold seasons, increasing the length of the supplementation period to two or more spermatogenic cycles, and increasing the sampling time point, to precisely identify at which stage of spermatogenesis CLA play a role, are recommended. Moreover, further investigation at the molecular level to evaluate the effect of CLA on sperm capacitation status, DNA integrity, and fertilization ability and investigating their effect on metabolites (e.g., Ca^++^) that play a role in semen quality are needed to draw conclusion on its effect.

## Figures and Tables

**Table 1:** Nutritional and chemical composition of Alfalfa hay, Reprotech Mature (AGFRI-Animal feeds^®^), and molasses meal (Molatek©).

Main Analysis	Unit	Alfalfa	Reprotech	Molasses
		Hay	Mature ^1^	Meal ^2^
Dry matter	% as fed	30.8	88.3	-
Crude protein	% DM	14.5	13	40
Crude fibre	% DM	34	6.03	100
Crude fat	g/kg	-	5.91	-
NDF *	% DM	48.2	-	-
ADF **	% DM	46.1	-	-
Lignin	% DM	9.1	-	-
Ether extract	% DM	1.9	-	-
Ash	% DM	8.7	9.28	-
Urea	g/kg	-	10	-
Calcium (Ca)	g/kg	-	9	13
Phosphorus (P)	g/kg	-	3.5	-
Ca: P Ratio	g/kg	-	2-3:1	-
Moisture	g/kg	-	11.7	150
Gross energy	MJ/kg DM	18.5	10.77	10.5

* NDF, Nitrogen detergent fiber; ** ADF, Acid detergent fiber. ^1^ AGFRI, Animal feed^®^, ^2^ Molatek, Ltd., South Africa.

## Data Availability

The data presented in this study are available on request from the corresponding author. The data are not publicly available due to privacy reasons, being the data from commercial bulls.

## Supplementary Materials

The following are available online at https://www.mdpi.com/2076-2615/11/2/483/s1, Table S1: Effect of CLA dietary supplementation on post-thawed bovine semen morphological analysis at meiosis phase by treatment group and sampling week; Table S2: Effect of CLA dietary supplementation on post-thawed bovine semen morphological analysis which affects nuclear (head) (LSM ± SE) at spermiogenesis phase by treatment group and sampling week; Table S3: Effect of CLA dietary supplementation on post-thawed bovine semen morphological analysis defects which affects the acrosome or tail (LSM ± SE) at spermiogenesis phase by treatment group and sampling week; Table S4: Effect of CLA dietary supplementation on bovine post-thawed semen on morphological analysis at maturation phase by treatment group and sampling week (LSM ± SE); Table S5: Effect of CLA dietary supplementation on post-thawed bovine semen morphological analysis for sperm cells with more than one defects in the same sperm cell (LSM ± SE) by treatment group and sampling week.

## Appendix A

Sample size:

To the best of our knowledge, this is the second study on dietary CLA supplementation in dairy bulls on sperm quality. The first study was published in 2016 by Karimi and collaborators [26] in the Reproduction of Domestic Animals Journal.

The number of animals needed was calculated by power analysis using the formula by Kaps and Lamberson [60]. According to the power analysis, 13 bulls were necessary and 14 were used.
