# Supplementary material for: Effects of Dietary Supplementation of Conjugated Linoleic Acids and Their Inclusion in Semen Extenders on Bovine Sperm Quality
Source: Animals (Basel). 2021 Feb 12;11(2):483. doi: 10.3390/ani11020483 (PMC7917996; doi:10.3390/ani11020483)
Supplement: Supplementary file 1 [file animals-11-00483-s001.pdf]

SUPPLEMENTARY MATERIAL

Table S1: Effect of CLA dietary supplementation on post-thawed bovine semen morphological analysis at meiosis phase by treatment group and sampling week.

Defect number*	Defect	Week	CTL	CLA
1	*Teratoid*	*-2*	0.28±0.10[a]	0.41±0.10[a]
		0	0.45±0.10[a]	0.25±0.10[a]
		4	0.64±0.10[a]	0.22±0.10[b]
		6	0.28±0.10[a]	0.20±0.10[a]
		10	0.16±0.10[a]	0.15±0.10[a]
		11	0.14±0.10[a]	0.15±0.10[a]
2	*Double*	*-2*	0.07±0.06[a]	0.05±0.06[a]
		0	0.21±0.06[a]	0.17±0.06[a]
		4	0.16±0.06[a]	0.14±0.06[a]
		6	0.12±0.06[a]	0.12±0.06[a]
		10	0.05±0.06[a]	0.10±0.06[a]
		11	0.19±0.06[a]	0.12±0.06[a]
3	*Macrocephalic*	*-2*	0.74±0.12[a]	0.88±0.12[a]
		0	0.45±0.12[a]	1.12±0.12[b]
		4	0.28±0.12[a]	0.69±0.12[b]
		6	0.21±0.12[a]	0.17±0.12[a]
		10	0.21±0.12[a]	0.31±0.12[a]
		11	0.26±0.12[a]	0.14±0.12[a]
4	*Microcephalic*	*-2*	0.36±0.11[a]	0.38±0.11[a]
		0	0.46±0.11[a]	0.45±0.11[a]
		4	0.24±0.11[a]	0.38±0.11[a]
		6	0.31±0.11[a]	0.28±0.11[a]
		10	0.65±0.11[a]	0.24±0.11[b]
		11	0.34±0.11[a]	0.31±0.11[a]
5	*Crested head*	*-2*	0.13±0.15[a]	0.23±0.15[a]
		0	0.49±0.15[a]	0.30±0.15[a]
		4	0.63±0.15[a]	0.11±0.15[b]
		6	0.34±0.15[a]	0.08±0.15[a]
		10	0.08±0.15[a]	0.08±0.15[a]
		11	0.18±0.15[a]	0.18±0.15[a]

a, b values (LSM±SE) with different superscript within rows are different ($P \leq 0.05$)

*Nöthling JO, and Irons PC 2008.

Table S2: Effect of CLA dietary supplementation on post-thawed bovine semen morphological analysis which affects nuclear (head) (LSM ± SE) at spermiogenesis phase by treatment group and sampling week.

Defect number*	Defect	Week	CTL	CLA
6	*Pyriform head*	*-2*	0.29±0.24 [a]	0.14±0.24 [a]
		0	0.29±0.24 [a]	0.29±0.24 [a]
		4	0.00±0.24 [a]	0.14±0.24 [a]
		6	0.14±0.24 [a]	0.57±0.24 [a]
		10	0.14±0.24 [a]	0.29±0.24 [a]
		11	0.57±0.24 [a]	0.29±0.24 [a]
7	*Narrow Head*	*-2*	0.25±0.15 [a]	0.20±0.15 [a]
		0	0.47±0.15 [a]	0.72±0.15 [a]
		4	0.20±0.15 [a]	0.58±0.15 [a]
		6	0.13±0.15 [a]	0.22±0.15 [a]
		10	0.32±0.15 [a]	0.32±0.15 [a]
		11	0.7 8±0.15[a]	0.25±0.15[b]
8	*Diadem*	*-2*	1.14±0.50 [a]	0.86±0.50 [a]
		0	0.00±0.50 [a]	0.00±0.50 [a]
		4	2.00±0.50 [a]	1.71±0.50 [a]
		6	0.29±0.50 [a]	0.57±0.50 [a]
		10	0.14±0.50 [a]	0.14±0.50 [a]
		11	0.14±0.50 [a]	0.43±0.50 [a]
9	*Narrow base*	*-2*	0.29±0.31 [a]	0.14±0.31 [a]
		0	0.29±0.31 [a]	0.43±0.31 [a]
		4	0.14±0.31 [a]	0.14±0.31 [a]
		6	0.43±0.31 [a]	0.57±0.31 [a]
		10	1.00±0.31 [a]	1.43±0.31 [a]
		11	1.29±0.31 [a]	1.14±0.31 [a]
10	*Abnormal base*	*-2*	0.14±0.15 [a]	0.14±0.15 [a]
		0	0.14±0.15 [a]	0.14±0.15 [a]
		4	0.29±0.15 [a]	0.29±0.15 [a]
		6	0.00±0.15 [a]	0.14±0.15 [a]
		10	0.00±0.15 [a]	0.00±0.15 [a]
		11	0.14±0.15 [a]	0.29±0.15 [a]
11	*Abnormal head*	*-2*	0.00±0.12 [a]	0.29±0.12 [a]
		0	0.14±0.12 [a]	0.00±0.12 [a]
		4	0.00±0.12 [a]	0.14±0.12 [a]
		6	0.29±0.12 [a]	0.00±0.12 [a]
		10	0.00±0.12 [a]	0.29±0.12 [a]
		11	0.00±0.12 [a]	0.14±0.12 [a]

a, b values (LSM ± SE) with different superscript within rows are different ($P \leq 0.05$)

*Nöthling JO, and Irons PC 2008.

Table S3: Effect of CLA dietary supplementation on post-thawed bovine semen morphological analysis defects which affects the acrosome or tail (LSM ± SE) at spermiogenesis phase by treatment group and sampling week.

Defect number*	Defect	Week	CTL	CLA
13	*Knobbed Acrosome*	*-2*	0.71±0.29 [a]	0.43±0.29 [a]
		0	0.57±0.29 [a]	1.14±0.29 [a]
		4	0.29±0.29 [a]	0.29±0.29 [a]
		6	0.14±0.29 [a]	0.29±0.29 [a]
		10	1.00±0.29 [a]	0.57±0.29 [a]
		11	0.29±0.29 [a]	0.71±0.29 [a]
14	*Stump tail*	*-2*	0.35±0.06 [a]	0.25±0.06 [a]
		0	0.11±0.06 [a]	0.06±0.06 [a]
		4	0.13±0.06 [a]	0.11±0.06 [a]
		6	0.08±0.06 [a]	0.27±0.06 [b]
		10	0.16±0.06 [a]	0.11±0.06 [a]
		11	0.16±0.06 [a]	0.23±0.06 [a]
15	*Pseudo droplet*	*-2*	0.00±0.13 [a]	0.00±0.13 [a]
		0	0.00±0.13 [a]	0.43±0.13 [b]
		4	0.00±0.13 [a]	0.00±0.13 [a]
		6	0.14±0.13 [a]	0.00±0.13 [a]
		10	0.00±0.13 [a]	0.00±0.13 [a]
		11	0.00±0.13 [a]	0.00±0.13 [a]
16	*Degenerate Mitochondria*	*-2*	0.14±0.20 [a]	0.14±0.20 [a]
		0	0.00±0.20 [a]	0.57±0.20 [b]
		4	0.57±0.20 [a]	0.43±0.20 [a]
		6	0.29±0.20 [a]	0.00±0.20 [a]
		10	0.29±0.20 [a]	0.14±0.20 [a]
		11	0.00±0.20 [a]	0.14±0.20 [a]
17	*Cork screw*	*-2*	0.00±0.17 [a]	0.00±0.17 [a]
		0	0.00±0.17 [a]	0.00±0.17 [a]
		4	0.00±0.17 [a]	0.00±0.17 [a]
		6	0.00±0.17 [a]	0.00±0.17 [a]
		10	0.14±0.17 [a]	0.00±0.17 [a]
		11	0.57±0.17 [a]	0.00±0.17 [b]
18	*Dag*	*-2*	3.00±0.84 [a]	1.43±0.84 [a]
		0	3.00±0.84 [a]	0.14±0.84 [b]
		4	2.57±0.84 [a]	0.43±0.84 [b]
		6	1.57±0.84 [a]	0.29±0.84 [a]
		10	1.57±0.84 [a]	1.57±0.84 [a]
		11	1.86±0.84 [a]	1.14±0.84 [a]
19	*Other midpiece defects*	*-2*	0.00±0.04 [a]	0.14±0.04 [b]
		0	0.00±0.04 [a]	0.00±0.04 [a]
		4	0.00±0.04 [a]	0.00±0.04 [a]
		6	0.00±0.04 [a]	0.00±0.04 [a]
		10	0.00±0.04 [a]	0.00±0.04 [a]
		11	0.00±0.04 [a]	0.00±0.04 [a]
20	*Coiled principle piece*	*-2*	0.21±0.08 [a]	0.15±0.08 [a]
		0	0.33±0.08 [a]	0.05±0.08 [b]
		4	0.14±0.08 [a]	0.20±0.08 [a]
		6	0.28±0.08 [a]	0.17±0.08 [a]
		10	0.21±0.08 [a]	0.25±0.08 [a]
		11	0.09±0.08 [a]	0.25±0.08 [a]
21	*Proximal droplet*	-2	0.40±0.26 [a]	0.63±0.26 [a]
		0	0.42±0.26 [a]	0.46±0.26 [b]
		4	1.25±0.26 [a]	0.60±0.26 [a]
		6	1.02±0.26 [a]	1.08±0.26 [a]
		10	1.06±0.26 [a]	1.60±0.26 [a]
		11	1.35±0.26 [a]	1.29±0.26 [a]

a, b values (LSM ± SE) with different superscript within rows are different (P ≤ 0.05)

* Nöthling JO, and Irons PC 2008.

Table S4: Effect of CLA dietary supplementation on bovine post-thawed semen on morphological analysis at maturation phase by treatment group and sampling week (LSM ± SE).

Defect number*	Defect	Week	CTL	CLA
22	*Midpiece reflex*	*-2*	2.30±0.81[a]	1.25±0.81[a]
		0	2.97±0.81[a]	0.96±0.81[a]
		4	3.39±0.81[a]	1.15±0.81[a]
		6	2.37±0.81[a]	1.89±0.81[a]
		10	1.99±0.81[a]	1.42±0.81[a]
		11	1.86±0.81[a]	1.13±0.81[a]
23	*Normally shaped Loose head*	*-2*	0.71±0.20[a]	1.29±0.20[b]
		0	0.62±0.20[a]	1.12±0.20[a]
		4	0.71±0.20[a]	0.62±0.20[a]
		6	0.54±0.20[a]	0.89±0.20[a]
		10	1.02±0.20[a]	0.67±0.20[a]
		11	1.00±0.20[a]	0.43±0.20[b]
24	*Fractured flagellum*	*-2*	0.93±0.14[a]	0.95±0.14[a]
		0	0.69±0.14[a]	0.69±0.14[a]
		4	0.55±0.14[a]	0.85±0.14[a]
		6	0.45±0.14[a]	0.47±0.14[a]
		10	0.41±0.14[a]	0.43±0.14[a]
		11	0.50±0.14[a]	0.21±0.14[a]
25	*Distal droplet*	*-2*	0.22±0.15[a]	0.26±0.15[a]
		0	0.17±0.15[a]	0.26±0.15[a]
		4	0.79±0.15[a]	0.11±0.15[b]
		6	0.81±0.15[a]	0.57±0.15[a]
		10	0.77±0.15[a]	0.92±0.15[a]
		11	0.91±0.15[a]	0.61±0.15[a]
26	*Degenerate acrosome*	*-2*	2.33±0.46[a]	3.02±0.46[a]
		0	2.00±0.46[a]	1.45±0.46[a]
		4	2.31±0.46[a]	2.05±0.46[a]
		6	2.52±0.46[a]	2.00±0.46[a]
		10	1.88±0.46[a]	1.83±0.46[a]
		11	1.88±0.46[a]	1.83±0.46[a]
27	*Bent midpiece*	*-2*	1.08±0.34[a]	0.97±0.34[a]
		0	1.56±0.34[a]	2.70±0.34[b]
		4	0.82±0.34[a]	1.75±0.34[a]
		6	0.30±0.34[a]	0.16±0.34[a]
		10	0.75±0.34[a]	1.09±0.34[a]
		11	0.80±0.34[a]	0.44±0.34[a]
28	*Bent principle piece*	*-2*	0.60±0.14[a]	0.20±0.14[b]
		0	0.63±0.14[a]	0.73±0.14[a]
		4	0.22±0.14[a]	0.90±0.14[b]
		6	0.27±0.14[a]	0.37±0.14[a]
		10	0.15±0.14[a]	0.35±0.14[a]
		11	0.49±0.14[a]	0.42±0.14[a]

a, b values (LSM ± SE) with different superscript within rows are different ($P \leq 0.05$)

*Nöthling JO, and Irons PC 2008.

Table S5: Effect of CLA dietary supplementation on post-thawed bovine semen morphological analysis for sperm cells with more than one defects in the same sperm cell (LSM ± SE) by treatment group and sampling week.

Combination of defects	Week	CTL	CLA
At least one nuclear (numbers 1 to 12) and one tail defect (numbers 13 – 28*)*	*-2*	0.24±0.16^{a}	0.30±0.16^{a}
	0	0.53±0.16^{a}	0.40±0.16^{a}
	4	0.51±0.16^{a}	0.21±0.16^{a}
	6	0.36±0.16^{a}	0.11±0.16^{a}
	10	0.41±0.16^{a}	0.11±0.16^{a}
	11	0.46±0.16^{a}	0.21±0.16^{a}
Two or more nuclear defects (numbers 1 to 12)*	*-2*	0.00±0.03^{a}	0.04±0.03^{a}
	0	0.12±0.03^{a}	0.04±0.03^{a}
	4	0.12±0.03^{a}	0.00±0.03^{b}
	6	0.05±0.03^{a}	0.00±0.03^{a}
	10	0.00±0.03^{a}	0.04±0.03^{a}
	11	0.10±0.03^{a}	0.04±0.03^{a}
Two or more tail defects (numbers 13 – 28)*	*-2*	0.21±0.10^{a}	0.19±0.10^{a}
	0	0.28±0.100	0.53±0.10^{a}
	4	0.24±0.10^{a}	0.34±0.10^{a}
	6	0.38±0.10^{a}	0.22±0.10^{a}
	10	0.14±0.10^{a}	0.10±0.10^{a}
	11	0.14±0.10^{a}	0.43±0.10^{b}
Normal	*-2*	83.29±5.83^{a}	86.71±5.83^{a}
	0	73.00±5.83^{a}	83.29±5.83^{a}
	4	82.86±5.83^{a}	85.14±5.83^{a}
	6	87.57±5.83^{a}	77.14±5.83^{a}
	10	86.57±5.83^{a}	83.00±5.83^{a}
	11	84.00±5.83^{a}	88.14±5.83^{a}

a, b values (LSM ± SE) with different superscript within rows are different ($P \leq 0.05$)

*Nöthling JO, and Irons PC 2008.
